# Health Transitions and the Rise of Modern Contraceptive Prevalence: Demand, Access, and Choice

**DOI:** 10.1111/padr.12662

**Published:** 2024-09-28

**Authors:** Jamaica Corker, Ann Biddlecom, Mohammad Jalal Abbasi-Shavazi, Alex Ezeh, Rodolfo Gómez Ponce de León

**Affiliations:** Independent Researcher, Seattle, WA, USA.; Independent Researcher, New York, NY, USA.; Vienna Institute of Demography, 1010 Vienna, Austria; Department of Demography, University of Tehran, 1411713118 Tehran, Iran; School of Demography, Australian National University, 2601 Canberra, Australia.; School of Public Health, Drexel University, Philadelphia, PA 19104, USA.; Pan-American Health Organization/World Health Organization (PAHO/WHO), Latin-American Center of Perinatology and Women’s Health (PAHO/CLAP), 11300 Montevideo, Uruguay.

## Abstract

Improvements in health and mortality, known as the health transition, played important roles in the rise of modern contraceptive prevalence across countries. We describe key mechanisms and selected research evidence that show how health transitions helped shape contraceptive transitions around the world. Mechanisms include how decreases in child mortality rates affect the motivation to use contraception and how the organization and expansion of health care affect key barriers to contraceptive use. Substantial increases in child survival resulting from health transitions increase demand for deliberate fertility regulation and contraceptive use. Improved access to primary health care, particularly maternal and child health services and expansion into rural communities, was associated with increases in modern method use. Country-specific policies that affected the organization and delivery of health care led to the dominance of particular modern methods in some countries. Empirical evidence is limited on how the quality of health care has affected aggregate level increases in modern method use. Using modern contraceptive prevalence to define the contraceptive transition reflects current data limitations but future research on the relationship of health transitions with macrolevel contraceptive prevalence trends may be able to incorporate more comprehensive aspects of how health transitions impact contraceptive choice and agency.

## Introduction

Contemporary contraceptive transitions have occurred in a context of historic declines in mortality, increases in life expectancy, and improvements in health, that is, “health transition.” It seems likely that contraceptive change, similar to but distinct from fertility change, has been related to the broader impacts of improvements in health and mortality. There are well-established arguments that high infant and child mortality undermines the motivation to restrict childbearing which, in turn, motivates contraceptive use. Beyond this more direct effect, there are possible effects on contraceptive use broadly of the expansion of health care that has been a key driver of declines in mortality and improvements in health seen across populations. To what extent this expansion of health care and other aspects of health transitions influence population-level transitions from low to high rates of contraceptive use, however, has been largely unexplored.

Demographic explanations of the rise in contraceptive prevalence are traditionally anchored to a framework of fertility decline (see Bulatao and Lee 1983). Much as the epidemiological transition helped to bring back a focus on what explains change in mortality levels and the distribution of causes of death (Caldwell 2001), a health transition framework broadens the theoretical focus on what explains contraceptive transitions from fertility decline per se to encompass mortality and health-related determinants. Moreover, exploring the impact of larger shifts in health care provision on contraceptive use gives due credence to the separate effects that broad improvements in health care have had from those due to family planning programs alone. It also opens up a reinterpretation of landmark intervention studies that are often cited for their family planning program impact.

In this paper, we focus on how health transitions contribute to changes in contraceptive use at the population level via changes in mortality levels and patterns of health care provision. The definition of contraceptive transition, shared by all papers in this volume, is the population-level transition of contraceptive prevalence from low levels (less than 10 percent use of modern methods) to high levels (more than 60 percent use of modern methods). A key revision we make to the definition of health transition, which we use to structure this paper and explore influences on the rise in contraceptive use across countries, is to characterize it as having two main components: change in mortality and cause of death patterns (i.e., the epidemiological transition) and change in how health care is organized and provided (i.e., the health care transition).

We describe key mechanisms linking health transitions with contraceptive transitions, distill existing empirical evidence, and offer a critical reflection on the utility of health transitions to explain and understand long-term changes in contraceptive use over time and space. Our priorities in organizing and analyzing evidence relating the health transition to contraceptive transitions are to focus on the quantitative dimension of contraceptive transitions—from low to high prevalence—and on evidence at the macro- and mesolevels of country and community. And while social change, including changes in health behavior, certainly occurs at and builds up from the individual level, the field of population studies lends itself to describing the impact of aggregated actions (e.g., cohorts, age structure changes) and larger societal shifts (e.g., period effects, changes over time). Analogous to the effect of maternal education on child health (Ewbank 1994), a focus at the individual level alone can miss the mechanisms of influence that community and health system contexts can have on contraceptive transitions.

To date, the weight of empirical evidence on the relationship between the health transition and contraceptive prevalence trends tends either to be in the opposite direction of our interest (e.g., how contraceptive use affects health outcomes) or focuses on contraceptive-specific services instead of the influence of health care provision more generally. Numerous studies have examined how contraceptive use, especially via the framing of it as a key health intervention (Jamison et al. 2018), has improved maternal and infant health by reducing high-risk pregnancies (Conde-Agudelo et al. 2012), improved birth spacing (Cleland et al. 2012), and reducing unintended pregnancies (Askew et al. 2017; Darroch Forrest 1994). There is also a long history of studies documenting the expansion of family planning programs, contraceptive technologies, supplies, and services, and analyzing associated changes in contraceptive prevalence (see Robinson and Ross 2007), including separate papers in this volume. While we acknowledge multidirectional causality in the relationship between health and contraceptive transitions and the value of family planning programs, this paper contributes to the wider literature by focusing instead on how broad changes in mortality and health care provision have affected increases in contraceptive use across countries.

## An overview of health transitions

What explains how major shifts in mortality and health care over time might shape contraceptive transitions? More than 50 years ago, Omran (1971) put forward a descriptive explanation of what he called the epidemiological transition—how the distribution of causes of death shift over time across countries—and major explanatory factors of these changes. He used evidence, primarily from a small set of developed countries over three centuries, to show the shift over time within a population from a dominance of infectious diseases as leading causes of death to a dominance of degenerative diseases and human-related (e.g., accidents) causes of death. Omran also identified ideal type stages and different pathways that countries have followed across these stages of the epidemiological transition and grounded changes in major causes of death and disease in a larger framework of population dynamics (i.e., mortality, fertility, and population growth). Other scholars expanded and revised Omran’s original framework to explain broader changes in health over time and space, including explicit attention to the social and economic determinants of health, conceptualizing the transition as a dynamic process instead of a time period-defined ideal type with a beginning and end point, and expanding the focus to evidence from low- and middle-income countries (Caldwell 1993; Defo 2014; Frenk et al. 1991; Santosa et al. 2014).

There is a wide range of determinants that shape population health over time through exposure to disease and individual susceptibility (Frenk et al. 1991). In an expanded framework of the epidemiological transition, Omran (1998) later included the macrolevel drivers of economic development and industrialization and other influential transitions categorized as lifestyle (e.g., personal hygiene behavior, nutrition) and education, health care, technology (though not clearly defined), and environment (e.g., sanitation, water, housing). Notably, this revised framework treated changes in health care as an associated characteristic of the epidemiological transition and not as a core part of the health transition per se.

Theorizing and evidence since Omran’s initial work also moved attention from national-level patterns and trends to inequities in health outcomes and interventions both across and within countries (e.g., by socioeconomic status, subnational geography, and population subgroup) (Frenk et al. 1991; Santosa et al. 2014); documenting and addressing health inequities as part of larger improvements in health are also increasingly reflected in international health goals and commitments, such as achieving universal health coverage, where essential health interventions (e.g., contraceptive services) should be accessible to all people (Jamison et al. 2018). Defo (2014) concisely summarized major critiques and expansions of health transition determinants as scholars who emphasized social, cultural, and behavioral factors behind changes in health across all countries (e.g., Caldwell and colleagues) and scholars who emphasized the health care system, including technologies and interventions (e.g., Frenk and colleagues). A systematic review of four decades of research on the epidemiological transition showed an increasing focus on social determinants of mortality and included macrolevel processes like climate change (Santosa et al. 2014). In the following sections of this paper, we examine how different aspects of health transitions contribute to changes in contraceptive use at the population level via changes in mortality levels and patterns and health care provision.

## Impact of mortality changes on contraceptive prevalence

Perhaps the most natural starting point for unpacking the influence of major shifts in mortality and health over time on changes in contraceptive prevalence is to examine how mortality decline—a key component of both the demographic and health transitions—impacts the motivation for deliberate fertility regulation that, in turn, drives contraceptive use. According to the demographic transition theory (Davis 1963; Notestein 1953) and the bulk of empirical evidence, as the demographic transition unfolds, fertility rates decline but only after initial declines in mortality rates. The empirical evidence on the epidemiological transition and fertility is less well-established but suggests impacts on fertility desires (including timing and assessment of risk) that accompany shifts in causes and distribution of mortality and morbidity that characterize the epidemiological transition. As both transitions unfold, they are likely to impact fertility desires and contraceptive behavior in related but distinct ways.

The onset of the demographic transition is marked by a population-level shift from high to low mortality rates, known as the mortality transition. Though there is some debate around the cause of the mortality decline historically (Casterline 2003), once mortality rates began to fall, they did so first among the younger age groups (Dyson 2010; Kirk 1996), with the most profound changes in overall mortality rates for young children and women of reproductive age (Omran 1971). This period of mortality decline is accompanied by increases in life expectancy, due primarily to improvements in infant and child survival, where mortality rates were concentrated pretransition. This in turn leads to substantial changes in the population age structure, with children accounting for a higher proportion of the population. Decreases in mortality at young ages also lead to greater numbers of surviving children and larger families, when birth rates remain high while mortality among infants and children falls. This results in what Dyson (2010) characterizes as a period of “disequilibrium,” during which populations often experience rapid growth during the time in which birth rates adjust to new and lower mortality rates.

The mortality decline in contemporary societies is driven by overall improvements in health and encompasses not only death rates but also improved health outcomes that have a direct effect on mortality (Johansson 2003). Yet while mortality transitions are often assumed to proxy health transitions, mortality rates can decline (e.g., due to a specific disease outcome) without corresponding broader improvements in health outcomes. Additionally, the effects of improvements in mortality and overall health on contraceptive use are often indirect. For example, the reduction in infant and child mortality, as a key determinant of contraceptive use, operates through the resulting lower demand for children and an increased desire to avoid unintended pregnancy (Lloyd and Ivanov 1988; Stover and Ross 2013).

Explanations of the increase in contraceptive prevalence are usually linked directly to a framework of fertility decline. We argue, however, that an examination of contraception transitions as separate from the fertility transition—similar to considering health transitions as distinct from the mortality transition—is warranted because increases in contraceptive use can follow, rather than precede, fertility declines. While contraceptive use is the largest contributor to fertility decline, other approaches to fertility regulation that are influenced by health transitions (e.g., abortion) have also played a role (Bongaarts and Hodgson 2022). Moreover, health transitions can have indirect effects that can both increase fertility (e.g., through overall improvements in health that increase fecundability) or decrease fertility (e.g., through better knowledge of and agency around contraceptive use, particularly for avoiding high-risk pregnancies).

There is broad agreement on the relationship between mortality and fertility decline, where declining mortality leads to lower fertility. The direct impact of decreases in mortality on subsequent changes in fertility, however, is an area of ongoing debate, particularly at the individual and household levels. Some scholars consider mortality decline alone to be a necessary and sufficient precursor for fertility decline, with the reduction in child mortality and greater numbers of surviving children leading directly to changes in fertility (Angeles 2010; Cleland 2001). At the individual level, this has been supported by evidence of the replacement hypothesis, where parents adjust fertility in response to child loss (Bousmah 2017; Hossain, Phillips, and LeGrand 2007; Nobles, Frankenberg, and Thomas 2015), and empirical findings related to the insurance theory, in which fertility may be precautionary and influenced by anticipated child mortality (Baynes et al. 2023; LeGrand et al. 2003). At the household level, downward pressure on fertility includes increased competition for family resources with more surviving children (Davis 1963) and the quality–quantity trade-off favoring greater investment in fewer children as survival to adulthood becomes more certain (Becker and Lewis 1973). Others, however, challenge the evidence around a direct relationship between a fall in death rates and a corresponding fall in fertility (Montgomery 2004; Palloni and Rafalimanana 1999; van de Kaa 1996; van de Walle 1986), instead attributing fertility decline to broader changes in attitudes and values related to family, including intergenerational relations (Caldwell 1976), valuing children as a source of personal fulfillment rather than economic value (Lesthaeghe 1980), having occurred without substantial mortality decline (Knodel and van de Walle 1979; Sathar 1992), or more directly influenced by levels of female education (Bongaarts and Hodgson 2022; Murtin 2013).

At the broader population level, though less directly measurable than at the individual or household level, psychological impacts related to comprehensive mortality declines can also play a role in changing fertility preferences as societies adjust to new mortality rates and patterns (see Lerch 2024 in this special issue for the impact of crisis-induced mortality). These psychological influences include decreased fatalism and an increased sense of personal control as mortality declines and becomes concentrated in older ages (Dyson 2010), as well as new perceptions around individual agency over behaviors related to future aspirations, including deliberate attempts at fertility regulation (Casterline 2003). Shifts in patterns of morbidity can also have effects on macrotrends in contraceptive prevalence but to a smaller degree; for example, the decreases in primary and secondary sterility for men and women that accompany health transitions can increase the demand for and use of contraception. Ultimately, despite the debate over the exact pathways and mechanisms through which mortality declines lead to changes in fertility (Mason 1997), there is widespread agreement that a preference for smaller families, directly or indirectly influenced by mortality change, is necessary for fertility decline (Bongaarts and Casterline 2018). It is this decline in the demand for children that in turn drives the demand to contracept. With the continued unfolding of the demographic and epidemiological transitions and declines in fertility, most people will spend the majority of their (reproductive) lives trying to avoid pregnancy, leading to profound reproductive and contraceptive behavior change that spans the life course.

## Patterns in health care provision and contraceptive use

The massive changes over time in the provision of health care, especially primary care, have also shaped contraceptive transitions by helping people put their preferences into practice. While the impact of mortality changes on contraceptive transitions operates via fertility desires and demand for contraceptives, the impact of health care on contraceptive transitions is mainly through the costs of contraceptive use (e.g., availability, access, and social acceptability). Lowering the barriers to contraceptive use is an important part of the story behind contraceptive transitions in many countries. For example, one study of time trends in modern contraceptive use in low- and middle-income countries found that much of what accounts for contraceptive change over time is implementation and not change in demand (Ibitoye, Casterline, and Zhang 2022), where increases in modern contraceptive use in recent decades were mainly due to increased satisfaction of demand for contraceptives rather than to changes in fertility preferences (see Yeatman and Sennott 2024 in this special issue for an in-depth treatment of the implementation of fertility desires into contraceptive use).

The health care transition lowers barriers to contraceptive use through several mechanisms. The first mechanism is improvements in the availability of and access to contraceptive information, counseling, and service provision independent of family planning programs. Prior to the formation and expansion of public health systems, people long used self-controlled methods of contraception to prevent pregnancy (such as withdrawal, rhythm, and early forms of condoms), while with modern methods (like the oral contraceptive pill, intrauterine device (IUD), implant, and sterilization) health clinics and health personnel have become crucial sources of contraceptive information and services. We acknowledge that contraception is unlike many other key health interventions in that some methods (e.g., condoms, oral contraceptive pills, or emergency contraception) are often widely available outside of a clinic setting and without the need for a prescription. However, the expansion of primary care often brought contraception as part of the core services provided, either explicitly as part of the package of national health care services (e.g., maternal and child health (MCH)), in coordination with family planning programs and nongovernmental organizations that were not formal parts of the national health care system, or both. Country case studies of the history of family planning illustrate these different approaches in detail (e.g., Chile, Thailand, and Turkey) and show the important role that health systems have played in the rising use of modern contraception over time (Robinson and Ross 2007).

A second mechanism linking health and contraceptive transitions is the legitimization of the public health impacts of contraceptive use and the medicalization of pregnancy prevention practices that affect, in turn, knowledge and social acceptability of using modern contraception. Public trust in health providers and, hence, the impact they can have in making contraceptive use acceptable, is also improved by the observable impacts of primary care in reducing local morbidity and mortality levels (independent of the health impacts of contraceptive use). We illustrate these mechanisms through different types of evidence that encompass both primary health care provision and contraceptive services or use.

A third mechanism is the quality of health care provided that can address, in part, key barriers to contraceptive use that go beyond access alone and that are often perpetuated outside of the health system, such as misinformation about contraception (e.g., methods can cause infertility), perceived and real health side effects of methods, and the acceptability of contraceptive use, especially for groups of people whose sexual behavior is often socially stigmatized (e.g., adolescents or unmarried women). While the evidence is limited on this mechanism at the country level over time, we describe several examples of its potential impact on increases in contraceptive prevalence.

In many countries, the expansion of primary health services into underserved areas, especially rural areas, with the assistance of community-based health workers and community engagement is an important part of the health care transition, both for increasing health care coverage and improving health outcomes. A recent review of hundreds of community-based, primary health care projects in low- and middle-income countries identified common strategies for effective implementation and showed the critical connections required between health systems and communities for effective delivery of care (Perry et al. 2017). While we do not focus on operational aspects of primary health care provision, we show through illustrative country examples how the health care transition has been an important part of contraceptive prevalence increases over time.

An early example is from Iran, where health care coverage during the 1940s and 1950s was sparse and a majority of rural areas did not have access to health services. Various attempts were made to modernize the health care system and reduce the inequality between rural and urban areas in the 1960s and 1970s, but these projects were suspended at the time of the 1979 Islamic Revolution. Two years after the revolution, however, the Ministry of Health implemented the health network system that had been planned by the previous regime. The central aim was to expand the access of people in rural and deprived areas of Iran to primary health care. By 2000, there were approximately 15,000 health houses [*khanah-e Behdasht*] throughout the country, each serving around 1500 people in a village and the surrounding satellite villages (Mehryar 2005). In rural areas of Iran, health houses followed an integrated health approach, with well-established health houses serving various functions, including health education, providing family planning, reproductive health, and disease control services, promoting environmental health, and collecting data and reporting on population health. Each health house had one or two health officers [*behvars*], one man and one woman, appointed by the Department of Health. Because *behvars* knew all or most of their clients, they could approach clients for follow-up treatment or family planning services and make referrals to higher levels of the health system. Importantly, the legitimization of family planning in particular was aided by support from religious leaders (Abbasi-Shavazi, McDonald, and Hosseini-Chavoshi 2009).

The establishment of the health network system and its extension to rural and deprived areas of the country was a key factor in the contraceptive transition in Iran (Abbasi-Shavazi, McDonald, and Hosseini-Chavoshi 2009; Salehi-Isfahani, Abbasi-Shavazi, and Hosseini-Chavoshi 2010). Following the implementation of this new system, child survival improved dramatically, with under-five mortality declining from approximately 95 deaths per 1000 live births in 1966 to 55 deaths per 1000 live births in 1996, influencing demand for smaller family sizes and fewer births among Iran’s population. From 1976 to 2000, the contraceptive prevalence rate rose from 37 percent to 72 percent (Abbasi-Shavazi, McDonald, and Hosseini-Chavoshi 2009; Salehi-Isfahani, Abbasi-Shavazi, and Hosseini-Chavoshi 2010). Recent model-based estimates for Iran over the past three decades (1990–2021) show a substantial increase in contraceptive prevalence among married or in-union women from 55 percent to 81 percent (United Nations Department of Economic and Social Affairs, Population Division 2022a), alongside a decline in under-five mortality from 57 to 13 deaths per 1000 live births (United Nations Inter-agency Group for Child Mortality Estimation (UN IGME) 2023). More recent concerns about low levels of fertility, however, have led to the introduction of pronatalist policies and changes in access to family planning programs in an attempt to reverse the fertility decline (McDonald et al. 2015), overestimating the role of contraceptive use in Iran’s fertility decline and limiting access to contraceptives with potential negative reproductive health consequences for women (Asadisarvestani and Sobotka 2023).

A more recent example of the impact of contraceptive use on expanded primary health care is Ethiopia’s Community Health Extension Program, implemented at a national scale starting in 2003. Initially targeting rural areas, it involved 38,000 full-time, salaried health extension workers (HEWs) and 17,000 health posts (Assefa et al. 2019; Olson and Piller 2013). Family planning was one of 17 health-related packages in the program and was under the family health domain, along with MCH, immunization, adolescent reproductive health, and nutrition (the three other program domains were disease prevention and control (e.g., malaria, HIV/AIDS, tuberculosis), hygiene and environmental sanitation, and health education and communication). A systematic review of program impacts showed that home visits by health extension workers were associated with increased preventive health behaviors and utilization of key health services across the board, including increased uptake of contraceptives (Assefa et al. 2019). Nationwide, modern contraceptive prevalence among married women aged 15–49 rose dramatically in just over a decade, from 6 percent in 2000 to 14 percent in 2005 and 27 percent in 2011 (mainly from increased use of HEW-distributed injectables) due, in part, to the intensive expansion of the primary health care program nationwide (Olson and Piller 2013). New model-based estimates for Ethiopia also highlight the stark changes from 2000 to 2021 in modern contraceptive prevalence among married or in-union women from 6 percent to 38 percent (United Nations Department of Economic and Social Affairs, Population Division 2022a) and decrease in under-five mortality from 140 to 47 deaths per 1000 live births (United Nations Inter-agency Group for Child Mortality Estimation (UN IGME) 2023).

From the other direction, community-based delivery of contraceptives also involves the provision of primary health care, information, and referrals for more than contraception alone. A systematic review of 56 studies in low- and middle-income countries showed that community health worker provision of contraceptives led to increased modern contraceptive use in more than 90 percent of studies, including 23 studies of community health workers who also provided other maternal and primary care services such as antenatal care and immunizations (Scott et al. 2015). Many of these studies, however, had incomplete information on the nature of noncontraceptive program information and services provided.

Very few studies have data over a long time period that permit disentangling health care and contraceptive care effects. One notable exception is a study in rural Nepal that used unique life history and health service and community history calendar data over approximately four decades and showed how changes in the availability of MCH services, *separate* from family planning services and other social and economic community changes (e.g., mass education, employment opportunities, transportation and market access, and media exposure), contributed to the rise of contraceptive use, from rarely used by couples to commonly used to limit childbearing (Brauner-Otto, Axinn, and Ghimire 2007). The provision of child immunization services increased the rate of contraceptive use to limit fertility, independent of family planning services, and other social and economic changes in the community. Moreover, if all three MCH services measured were available, contraceptive use was substantially higher, net of family planning and other social and economic contextual measures, than if none were offered. These effects were robust across different measures of availability across space (nearest provider, fixed radius catchment area, and a geographically weighted summary measure of service provision).

At the household level, there are also positive knock-on effects from increased utilization of maternal health services on subsequent contraceptive use. For example, an analysis of reproductive health calendar data from household surveys, which provide detailed timing of behaviors, in 43 countries across six regions from 2003 to 2013 found a strong positive correlation between the use of maternal health care (measured as antenatal care and facility-based delivery for the most recent birth) and contraceptive use in the postpartum period (Winfrey and Rakesh 2014, Table 9). Only 7 percent of women who had no antenatal care (ANC) were using contraception at three months postpartum compared to 14 percent among women with one to three ANC visits, 28 percent of those with four to six ANC visits, and 38 percent with seven or more visits. Similar results were obtained in an analysis of calendar data and facility-based delivery (Tsui, Brown, and Li 2017), although neither study controlled for individual or household characteristics that may have affected the associations.

## Health care provision and impact on specific method prevalence

The nature and form of health care provision have also shaped the types of methods that can come to dominate during a contraceptive transition, beyond—or even despite—formal family planning policies aimed at expanding contraceptive options. In Brazil from 1964 to 1980, fertility was declining with rising preferences for smaller family size during a period when there was no public family planning program, limited available contraceptive options, and care provision under a military government that focused on hospital-based curative care (Cavenaghi and Alves 2019; Potter 1999). With regulations that made it difficult to provide and be reimbursed for female sterilization, doctors offered surgical sterilization as part of cesarean section delivery during a time when cesarean deliveries were increasing substantially throughout the country because the cesarean cost reimbursement rate was greater than for vaginal delivery. As a result, in the 1980s nearly two in three sterilized women received the procedure during a cesarean delivery (Potter 1999). Yet even after the financial incentive for cesarean sections had been removed and national policies were implemented to expand contraceptive options, female sterilization continued to increase from 27 percent in 1986 to 40 percent in 1996 among married or in-union women of reproductive age, with 60 percent of sterilizations performed during a cesarean section, before declining to just under 30 percent in 2006 and 2013 (Cavenaghi and Alves 2019; Potter 1999). In tandem, modern contraceptive prevalence among married or in-union women in Brazil increased from 57 percent in 1986 to 70 percent in 1996, then increased slightly and held steady at 78 percent in 2006 and 2013 (Cavenaghi and Alves 2019), showing how female sterilization played a key role in the rapid increase in modern contraceptive prevalence in Brazil.

Another example of the impact of health care provision on the uptake of a particular method comes from Indonesia and the Village Midwife Program, which expanded access to primary care to improve MCH, especially for economically disadvantaged and rural communities, and provided an additional source for modern contraceptives (Shankar et al. 2008). The timing was at a point in the country when contraceptive use was already fairly common, with 47 percent of married women of reproductive age using a modern method in 1991, mostly pills (15 percent) and IUDs (13 percent) (United Nations Department of Economic and Social Affairs, Population Division 2022b). Approximately 54,000 midwives were deployed across the country within the first seven years of operation (1989–1997), and they were expected to financially support their work after three years. Studies show the program increased the use of maternal health services (antenatal care, taking iron supplements, skilled birth attendant at delivery) (Frankenberg, Suriastini, and Thomas 2005) but had no impact over 14 years on modern method use (Weaver et al. 2013). However, this expansion of health care did influence changes in specific methods used, primarily a substantial rise in the use of injectables (Weaver et al. 2013), likely fueled in part by the need for midwives to be financially self-sustaining and the incentive of a consistent payment flow from injectables compared to long-acting methods like IUDs, implants, and sterilization (Hull and Mosely 2009).

The cases of Brazil and Indonesia indicate that the expansion of health care can lead to distinctive change in the method mix but not necessarily to increases in overall rates of modern contraceptive use. Additionally, both overall contraceptive prevalence and method-specific use have been heavily influenced in contexts with a history of coercive family planning programs, for example, India (where sterilization is dominant) and China (where IUDs dominate the method mix), although we consider such situations to be outside the scope of this paper as they result primarily from direct programmatic interventions rather than other aspects of overall health transitions. As noted earlier, there are other case studies that descriptively show the intertwining of national health care systems and, in this case, the dominance of particular methods in contraceptive trends over time (Robinson and Ross 2007).

## Quality of health care and contraceptive prevalence trends

Much of the evidence on health care and contraceptive use described thus far has focused on the expansion of health care and how it improved access to contraceptive services. The quality of health care provided—that is, how and what is provided—also plays an important role in reducing barriers to contraceptive use and, as a result, has the potential to increase modern contraceptive prevalence over time. Indeed, neglecting the quality of care in health programs that rush to get an intervention out to the public can have unintended negative consequences for health care utilization and outcomes (see Robinson and Ross 2007 for the case of family planning programs).

As health care transitions take off, and more people gain access to core health services, the quality of services provided assumes more importance for continued increases in health care utilization and for addressing, in part, common barriers to utilization beyond access per se. Omran’s (1998) updated conceptual framework of the epidemiological transition calls attention to the stresses that health care systems experience in providing quality health services when faced with rising demand for health care and an increased overlap in health problems (i.e., acute and chronic illnesses). In other words, as access to health care improves, there is an accompanying need to prioritize the quality of health care to further improve positive health outcomes from health service use.

An illustration of this shift is in the provision of obstetric services over time. Souza et al.’s (2014) description of the obstetric transition, where countries move from high maternal mortality towards the elimination of maternal deaths, shows that in the early stage when maternal mortality is at high levels, more women start seeking care at health facilities and a critical issue is thus access to care. As a higher proportion of expectant parents reach health facilities, the quality of care provided then becomes a major determinant of maternal and newborn health outcomes, and this is especially pertinent for health facilities facing increased demand. A cross-sectional application of this framework to a large maternal and perinatal health database from 29 countries across three regions supported the correlation between maternal mortality and morbidity levels and the need for a shift from improving access to improving the quality of health services (Chaves et al. 2015).

While the substantive argument for the impact of quality of care on modern contraceptive prevalence trends is compelling, the research evidence on this link is quite limited. Many studies that examine the impact of quality of services on contraceptive use are cross-sectional (e.g., Arends-Kuenning and Kessy 2007) and cannot tell us the degree to which quality of care has shaped overall contraceptive prevalence trends in a country. We do know, however, that some common barriers to contraceptive use can be addressed—or exacerbated—by the quality of health care delivery, including provider bias, misinformation about method safety, and user concerns or discomfort with side effects.

In her foundational framework on quality of contraceptive care, Bruce (1990) showed how high-quality care could address distinct barriers to contraceptive use, for example, by expanding the meaningful choice of contraceptive methods offered to ensure a range of methods to meet people’s different needs (e.g., for spacing or limiting births, preventing sexually transmitted infections (STIs) or preferences for nonhormonal contraception). High-quality contraceptive counseling—including explaining the risks and benefits of different methods, how to use them effectively, what to do if people experience side effects, and appropriately responding to people’s questions and concerns—plays a key role in effectively addressing the potent barriers to contraceptive use of health concerns and misinformation. High-quality services also enable continuity of care to appropriately address method-specific problems and facilitate method switching rather than discontinuation for people in need, which can impact overall levels of contraceptive prevalence.

Issues of quality of care are also related to systemic inequities in contraceptive access and use that could impact the speed of contraceptive transitions. For example, provider bias is a common barrier that adolescents and young adults face in the health system when health providers refuse or limit contraceptive information and services because they disapprove of premarital sexual activity or they incorrectly believe some methods, like long-acting hormonal methods or the IUD, are inappropriate for young women who have not started childbearing (Chandra-Mouli et al. 2014). Such bias not only deprives young people of accurate information and support for making health decisions, but from a longer-term perspective, this barrier sets large cohorts of people with a built-in health disadvantage as they enter their reproductive years and may unnecessarily slow the uptake and continued use of modern methods over time. Ultimately, while the substantive argument for the impact of quality of care on trends in contraceptive prevalence is compelling, the dearth of research evidence limits our ability to make direct links between quality of health care provision and contraceptive transitions.

## Revisiting well-known studies on family planning program impact

Situating increases in contraceptive prevalence within a broader health transition framework may lead to a reassessment of the impact on contraceptive use attributed to targeted family planning programs. Two rigorous and oft-cited studies of family planning program impacts from Bangladesh (Matlab) and Ghana (Navrongo), in particular, merit revisiting to call attention to the MCH care elements that were important parts of the programs’ content and design but that often receive short shrift in descriptions of the studies when the focus is on contraceptive use (e.g., Bongaarts and Hodgson 2022). The Matlab Family Planning and Health Services Project began in 1977 in Bangladesh with a family planning intervention and a set of MCH services, with further MCH services (e.g., oral rehydration therapy, tetanus and measles vaccination, and antenatal care) that were added in subsequent stages across select villages (Phillips et al. 1984). Contraceptive prevalence rose from below 10 percent in 1977 to 36 percent by mid-1983. While there were no consistent, positive effects of intensified MCH services on contraceptive uptake, the study authors noted that the first set of MCH services offered at the same time as family planning was an important part of the dramatic increase in contraceptive prevalence over time and was, in fact, a relatively substantial set of services that included, in their words,

…(1) a family welfare center program with curative care and contraceptive backup services provided by trained resident paramedics, carefully organized referral links from the field with meetings to ensure coordination between the welfare center and the community health worker, and basic pharmaceuticals free of charge; (2) a trained female work force with basic health knowledge so that health themes and referral could be incorporated into motivational discussions, thereby lending credibility to discussions of family planning; and (3) basic MCH outreach to include discussions of nutrition, sanitation, and child care and dispensation of aspirin, vitamins, and iron tablets.

(Phillips et al. 1984, 158)

In short, the program well known for the positive impact of contraceptive services was, in its design and rollout from the start, a wider set of MCH services offered at the same time as contraceptive services, and that were an important part of the dramatic increase in contraceptive use in the exposed villages.

The Navrongo Community Health and Family Planning Project, fully launched in 1996 in a remote, rural area in northern Ghana with high mortality and fertility and very low modern contraceptive prevalence (under 6 percent among married women), focused from the start on expanded access to primary care in addition to the provision of family planning services (Debpuur et al. 2002), even though the motivating research hypothesis and outcomes were about reproductive change. Two of the project’s three treatment cells consisted of the provision of basic primary health care through nurses newly located within villages and family planning services, both of which were added to clinic-based services already in place and part of regular public health services. Community-based nurses improved access to primary care such as treatment for respiratory illnesses, malaria, and diarrhea, and improved vaccination coverage. These community-based nurses led to accelerated gains over time in child survival versus comparison areas (Phillips, Bawah, and Binka 2006) and increased women’s desire to limit fertility (Debpuur et al. 2002), though the provision of primary care services cannot be disentangled from the family planning services that nurses also provided. The impact on modern contraceptive use was only evident when combined with community health volunteers, whose actions addressed the social costs of contraception and involved outreach to men. Other case studies from different countries and regions that describe the intertwining of family planning programs with expansions in MCH services or primary health care (Robinson and Ross 2007), though without the same rigorous testing as these two foundational studies, pose similar challenges to distinguishing between successful aspects of targeted family planning programming from the influences of broader changes in patterns of health care provision.

## Implications

From a conceptual standpoint, the impact of morbidity and mortality and health care provision on contraceptive use aligns closely with the three preconditions of fertility decline by Coale (1973) and adapted by Lesthaeghe and Vanderhoeft (2001): people’s desire to regulate their fertility, their access to contraception (e.g., knowing about methods and having reasonable access), and their favorable attitude to using contraception (e.g., social acceptance, fear of side effects not a barrier). The effect of improvements on health and reductions of mortality and morbidity illustrates the impact of people’s desire to use contraception through their increased motivation to prevent unintended pregnancy, especially high-risk pregnancies or a pregnancy soon after the birth of a child. The impact of health care provision aligns closely with satisfying demand for contraception by addressing barriers to using contraception, specifically people’s access to and attitudes about using modern contraceptives. Easterlin (1975) similarly described costs to “fertility regulation” in ways related to broader impacts of changes in health care provision, categorizing the costs as either objective costs (market-based factors or “access,” such as the time and money required to learn about and use specific methods) or subjective (attitudes). He recognized that both sets of barriers are shaped, in turn, by broader societal attitudes about fertility regulation and specific techniques as well as the availability of information and the range of specific techniques and their prices.

We acknowledge that there are important implications of broader aspects of the health transition for contraceptive transitions that we do not directly address. To manage the scope of evidence and arguments we explored in this paper, we necessarily excluded in-depth treatment of other facets of health transitions that could also affect contraceptive use trends. For example, public trust in health care institutions and modern medical interventions, changes in public attitudes and health behaviors that increasingly emphasize prevention or normalize health practices (e.g., taking pills or receiving injections), and public health promotion activities (e.g., campaigns to reduce unintended pregnancy) that may have direct or indirect effects on people’s motivation to use contraception and seek contraceptive services. Instead, in seeking to highlight the underappreciated role of health transitions on contraceptive transitions, we focused on identifying the main mechanisms through which health transitions can directly impact contraceptive transitions rather than on cross-cutting areas or secular factors that interact with health transitions and also influence changes in macrolevel contraceptive trends. One of these secular factors is education, particularly women’s education, and its relationship with both health-seeking behavior more generally and contraceptive use specifically. Increases in women’s education globally have played and will continue to play a key role in shaping contraceptive prevalence trends (Bongaarts and Hodgson 2022), but education also interacts with the health transition, particularly around people’s willingness to seek and demand health care services. Education not only leads to more information, awareness, and health-related behavior change but can also influence ideals about quality of life, including the demand for children and the demand for high-quality health care products and services. Greater awareness of and agency over preventative care is a by-product of both improved education and health transitions and extends to contraceptive use as a form of preventative care.

Looking forward, changes underway related to contraceptive access and use could make lessons from past links between health care and contraceptive transitions less informative for understanding the relationship between these transitions in the future. First, the push to include family planning and contraception as part of universal health coverage may lead to greater equity and more balanced access to contraception across different population groups compared to the past, leading to different patterns of growth in contraceptive prevalence. This will be increasingly relevant as lower lifetime desired fertility across different population groups means a growing number of people will want to spend most of their reproductive lives using contraception. Second, the growth in digital communication and new contraceptive technologies, with an increased emphasis on self-care and demedicalization of contraception (e.g., self-injectable and emergency contraception), continues to move control over contraceptive choice out of the health care system and towards users. This could lead to less reliance on formal health systems for information on and access to modern methods and increased use of the private sector, including direct-to-consumer services and delivery. Third, further advances in contraceptive technology, including new methods or key improvements in existing methods, could impact the pace of change in future contraceptive transitions in settings where contraceptive use remains low. Fourth, greater knowledge of the noncontraceptive benefits of many hormonal methods—for reduced endometrial or ovarian cancer risk (WHO 2012), relief for menstrual pain and related disorders, or even acne treatment (Maguire and Westoff 2011)—is also contributing to the use of modern methods for reasons other than preventing pregnancy. In addition to increasing levels of use, the use of modern methods for noncontraceptive benefits may also change the profile of contraceptive users; for example, approximately 14 percent of pill users in the United States choose pills exclusively for noncontraceptive reasons, of whom half have never had sex (Kavanaugh and Anderson 2013). Fifth, future research may not be restricted to using contraceptive prevalence as the outcome. Like all papers in this volume, our aim is to understand past patterns in changes in population-level contraceptive prevalence. There are, however, additional important aspects related to contraceptive use and choice to monitor over time, including the choice not to use contraception, preferences around specific methods, and method continuation and switching, among others. Our focus here on modern method use, while informative, is not meant to promote or privilege modern use over other important aspects of choice and decisions around fertility regulation that may be more directly addressable in future research with greater data availability on noncontraceptive use outcomes.

Finally, there are broader implications of this paper for assessing macrolevel changes in contraceptive use over time and across countries, including for evaluation of the impact of family planning programs on contraceptive change net of overall changes in health care provision. Our critical reflection on the rise of contraceptive prevalence against the backdrop of broader health transitions leads to questions about whether increased contraceptive use attributed to family planning programs might actually be due to broader healthcare expansion. Situating landmark studies of family planning programs, such as the Matlab and Navrongo studies, within a health transition framework may prompt a reassessment of how broad shifts in health care provision, as opposed to specific family planning interventions, have contributed to significant increases in contraceptive use. This should not, however, be interpreted as advocating for integrated approaches to health care and contraceptive programming, though such integrated approaches may have the largest impact in some settings. Instead, it is a call to more deliberately disentangle which factors most directly influence contraceptive prevalence change in particular contexts in order to more effectively target family planning programs, policies, and funding where they will have the greatest impact on meeting contraceptive needs.

## Conclusion

The health transition played an important role in the rise of modern contraceptive prevalence across countries. The first way is through the substantial reduction in infant and child mortality and the resulting increase in demand for fertility regulation and contraceptive use. Substantial declines in mortality in the youngest age groups, as part of the health transition, lead to increases in demand for smaller families and thus deliberate attempts at fertility regulation, either as a direct result of pressures from a greater number of surviving children within families or via broader changes in attitudes and values around children that accompany the demographic transition.

The second way is through the expansion of health care and consequent reduction in key barriers to contraceptive use. The health care transition increases contraceptive prevalence over time by increasing the availability of contraceptives and services as part of health care expansion, reducing knowledge and social acceptability barriers to use through the legitimization of contraception as an approved medical intervention that has positive health impacts, and reducing other barriers to use (e.g., misinformation) through improvements in the manner and content of health care delivery. Improved access to primary health care, particularly as part of MCH services and expansion into rural communities through community-based care, is also associated with increases in modern method prevalence. Context- and country-specific policies and incentives affect the organization and delivery of health care in ways that can influence not only the timing but also specific aspects of the contraceptive transition, for example, the dominance of particular modern methods in some countries. Though we are able to theorize about mechanisms through which high quality of care can influence the rate and characteristics of contraceptive transitions, the lack of empirical evidence limits what we can say about how the quality of health care, in particular, has affected increases in modern method use at the aggregate level over time.

The health transition framework evolved from describing health as a product of development to health as a fundamental part of development, situating health as a right, and focusing public health priorities on addressing health inequities. This will continue to change how the health transition is conceptualized, measured, and understood. Likewise, the continued efforts to move away from defining and measuring contraceptive use as the primary outcome of interest related to family planning towards more comprehensive rights-based approaches and measures (Gruskin et al. 2017; Holt et al. 2023; WHO 2015) will redefine the future study of the relationship between health transitions and contraceptive transitions. In contrast to many health areas and interventions impacted by health transitions, 100 percent of targets or objectives (e.g., eradicating polio or eliminating preventable maternal mortality) are not appropriate or acceptable for contraceptive use. Family planning and contraceptive use must respond to individual choices and changing levels of demand and preferences, with the decision to use (or not to use) contraception considered to “constitute resources, rather than ends in themselves” (Coates and Allotey 2023).

Looking ahead, future research on contraceptive transitions could expand to include qualitative aspects of contraceptive transitions such as the alignment of methods with user needs and preferences (e.g., access to emergency contraception), equity in access to contraception across different groups and contexts (e.g., people living in poverty, adolescents, people with disabilities, or migrants and refugees), or other important aspects of contraceptive dynamics such as reasons for use and nonuse, discontinuation and method switching. Our focus here on past patterns of modern contraceptive use to define the contraceptive transition reflects the limits in existing data beyond measurements of contraceptive use. While an improved understanding of trends in contraceptive use has value for understanding the relationship between population-level health and contraceptive transitions, better and different data moving forward will enable improved definitions for and study of contraceptive transitions, ultimately expanding our understanding of the relationship of health transitions not only with levels of contraceptive use but also with important aspects of contraceptive choice, agency, and rights-based care.
